# Optimal Duration of Treatment for HCV Genotype 1 Infection in Slow Responders: A Meta-Analysis

**DOI:** 10.5812/kowsar.1735143X.721

**Published:** 2011-08-01

**Authors:** Seyed Moayed Alavian, Seyed Vahid Tabatabaei, Bita Behnava, Nastaran Mahboobi

**Affiliations:** 1 Baqiyatallah Research Center for Gastroenterology and Liver Diseases, Baqiyatallah University of Medical Sciences, Tehran, Iran

**Keywords:** Hepatitis C virus, Genotype, Treatments

## Abstract

**Background:**

A head-to-head comparison of the 72-week and 48-week anti-HCV therapies in slow responders with genotype 1 infection has been performed in several randomized clinical trials (RCTs).

**Objectives:**

This review aimed at summarizing and pooling the results of these studies.

**Materials and Methods:**

RCTs that had evaluated the 72-week vs. 48-week anti-HCV therapy (peginterferon and ribavirin) in slow responders with HCV genotype 1 infection were systematically identified. A meta-analysis was performed using the random effects model. Heterogeneity in results was assessed on the basis of the Q statistics, and publication bias was evaluated by using Harbord’s modified test. The end point was set as a sustained virological response (SVR).

**Results:**

Data of 1206 subjects were retrieved from 7 studies. A total of 631 patients had received extended therapy. Slow virological responders who received the 72-week therapy had a significantly higher probability of achieving SVR than their counterpartswho received the 48-week therapy [RR = 1.44 (95% CI, 1.20–1.73)]. With regard to publication biases, the heterogeneity in funnel plots was not significant (P = 0.19, I2 = 30%, PHarbord = 0.1).

**Conclusion:**

Our meta-analysis showed that the 72-week therapy with peginterferon and ibavirin is significantly superior to the standard 48-week therapy in slow responders th HCV genotype 1 infection.

## 1. Background

Hepatitis C virus (HCV) infection is a major cause of chronic liver disease, cirrhosis, and hepatocellular carcinoma (HCC) worldwide, and may necessitate liver transplant in the patients with these diseases [1][2][3]. In 80% of the cases with HCV infection, the infection progresses to a chronic state, and in approximately 20% of the cases, chronic HCV infection may lead to cirrhosis [4]. Achievement of a sustained virological response (SVR) to antiviral therapy prevents the progression of fibrosis and cirrhosis and decreases the risk of HCC, thereby improving the survival rate of patients. Therefore, antiviral therapy is an important treatment option in the clinical management of these patients [5][6][7][8][9].

Currently, the standard therapy for HCV genotype 1 and genotype 2/3 is administration of regimens of peginterferon alpha and ribavirin for 48 weeks and 24 weeks, respectively. Patients with rapid virological response (RVR; undetectable HCV-RNA level at week 4 of therapy) have 80–100% likelihood of achieving SVR, whereas those who do not achieve an early virological response (EVR; undetectable HCV-RNA level at week 12 of therapy or less than 2-log decrease in RNA level compared to a pretreatment RNA level) have only an 8% chance of achieving SVR [10][11]. Since viral kinetics play an important role during therapy, several studies have attempted to evaluate individualized anti-HCV therapy on the basis of the patients’ virological response instead of HCV genotype alone. In rapid virological responders with HCV genotype 2/3 infection, therapy for a duration shorter than that of the standard therapy was as effective as the standard therapy; however, in cases of HCV genotype 1 infection, different results have been obtained [12][13]. Several studies have evaluated the effects of extending the therapy in slow responders.

## 2. Objectives

In this review, we aimed to summarize and pool the results of these studies to determine the optimal duration of treatment for HCV genotype 1 infection in slow responders.

## 3. Materials and Methods

### 3.1. Search Methods for Identification of Studies

We performed an electronic search of Medline, EMBASE, Scopus, Cochrane Central Register of Controlled Trials, and ISI Web of Knowledge (SV Tabatabaei, B Behnava). The keywords we used were different combinations of “hepatitis C virus” or “HCV” with the following terms: “slow responders”; “72-week”; “extended therapy”; “rapid virological response” or “RVR”; “early virological response” or “EVR”; “peginterferon alpha-2a”; “peginterferon alpha- 2b”; and “ribavirin” or “RVB” or “RBV”. In addition, terms such as “Pegasys,” “Pegintron,” “Rebetol,” and “Copegus” were used. We examined the references cited in the reviewed papers to find other relevant studies. Temporal limits were not applied in our search strategy.

### 3.2. Data Collection and Analysis

All citations were imported to an EndNote library. Further, the titles and abstracts were screened by 2 investigators (SV Tabatabaei, B Behnava) who were unaware of each other’s study selection. Full texts of all the selected reports were retrieved and assessed according to our predefined inclusion and exclusion criteria. Data from studies that met our criteria were extracted by 2 investigators separately and rechecked by a third investigator. Data of the outcomes of treatment were tabulated according to the treatment regimen in Excel spreadsheets. Predefined assumptions and the decision to include or exclude a study were made and agreed by all authors before the meta-analysis. Data on the characteristics of patients and studies were summarized by using standard questionnaires that included the first author’s name, name of journal, methodology of randomization, allocation concealment, publication year, and sample size in each treatment arm as well as viral loads, liver histologies, frequencies of genotypes, and SVR.

### 3.3. End Point of Interest

The end point for comparison of efficacy was SVR, defined as undetectable HCV-RNA level 6 months after treatment cessation.

### 3.4. Inclusion and Exclusion Criteria

Randomized clinical trials (RCTs) on patients with chronic HCV infection who were seronegative for HIV and hepatitis B virus (HBV) infection were included if the patients in the trials [1] were randomized to receive either peginterferon alpha-2a 180 μg/kg per week or peginterferon alpha-2b 1.5 μg/kg per week plus weightbased ribavirin during either the standard (48 weeks) or extended therapy (72 weeks), [2] had detectable HCVRNA level at week 4 of the treatment, [3] achieved at least a 2-log decrease in HCV-RNA levels from the baseline to 12 weeks or undetectable HCV-RNA at 24 weeks, [4] had genotype 1 infection, and [5] were diagnosed with chronic HCV infection on the basis of detectable HCV-RNA level and infection for at least 6 months. We included articles from all languages that met these criteria. Furthermore, our meta-analysis included studies that had patients with a history of treatment and for whom study dose modification and administration of growth factors and antidepressants were performed. Even the studies that included patients with infections caused by other HCV genotypes or both rapid and slow responders were included if they provided enough data for reanalysis of the data for the subset of slow responders with HCV genotype 1 infection. Studies were excluded if the patients: [1] had decompensated liver disease, [2] were seropositive for HIV or HBV markers and, [3] had significant co-morbidities such as decompensated liver disease, autoimmune diseases, hemoglobinopathies, and chronic kidney disease, [4] received low-dose ribavirin or peginterferon during some parts of their treatment period, and [5] had negative PCR findings at week 4 of treatment.

### 3.5. Methodological Quality Assessment of RCTs

The methodological quality, defined as the confidence that the design and reporting of a trail will restrict the possibility of a bias in intervention comparison, was evaluated as previously reported [14]. The only difference was that the blinding of patients or investigators was not feasible in the current study because of the different treatment durations. Allocation sequence generation and concealment were obtained as measures of bias control. The allocation sequence generation was considered adequate if it was obtained through a tableor computer-generated random numbers. The allocation concealment was considered adequate if the patients were randomized by a central independent unit or by using serially numberedopaque sealed envelopes.

### 3.6. Data Synthesis

All analyses were performed using the Mix 2.0 professional software for meta-analysis with Excel [15]. Data on all patients were included on the basis of intention-to-treat principle, irrespective of compliance or follow-up. To manage the missing data, we performed a worst case scenario analysis. However, since we had a positive outcome (virological response), all missing data were considered as data from the non-responders. The results are presented as relative risk ratio (RR) with 95% confidence interval (CI). The meta-analysis was performed using the DerSimonian and Laird random effects model. The random effects model provides a highly conservative estimate of significance. This model operates under the assumption that the included studies are only a random sample of all studies. Hence, heterogeneity between individual studies will result in a wider CI of the summary estimate. The summary estimate was calculated as an average of the individual study results weighted by the inverse of their variance by using the DerSimonian and Laird random effects model [16]. The estimate of heterogeneity was obtained from Q statistics. The study findings were considered heterogeneous if the resultant P-value was less than 0.1 [17]. Furthermore, I2 was used to provide a measure of the degree of inconsistency between the results of the studies. Its value describes the percentage of total variation across studies that is caused by heterogeneity rather than chance. The I2 value lies between 0% and 100%. A value of 0% indicates no observed heterogeneity, whereas larger values indicate increasing heterogeneity [18]. Publication bias was assessed by testing for funnel plot asymmetry by using Harbord’s modified test. Several statistical tests can be used to evaluate funnel plot asymmetry. However, among these tests, a regression-based approach is most appropriate for detecting significant publication bias. For analyzing binary outcomes, the Harbord’s modified test is preferred over Egger’s test because Harbord’s modified test has a lower likelihood of showing false-positive results due to the mathematical association between logRR and its standard error [19].

### 3.7. Transparency Declarations

The authors declare that there are no conflicts of interest, financial or otherwise regarding the contents of this review. Furthermore, this meta-analysis was not supported by any pharmaceutical company, government agency, or grants from other sources.

## 4. Results

### 4.1. Results of the Search

**Figure 1:**
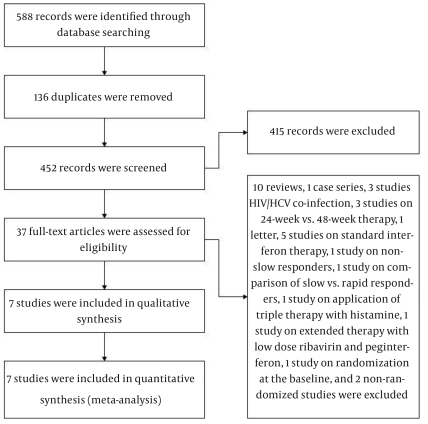
Search Result Analysis

Our search strategy yielded 452 unique citations. Figure 1 shows our search analysis protocol. We discarded 415 nonrelevant records and retrieved full texts of 37 potentially relevant papers. We excluded: 10 reviews regarding the optimal treatment of infections caused by different HCV genotypes [20][21][22][23][24][25][26][27][28][29], 1 case series with 9 subjects [30], 3 studies that evaluated 24- vs. 48-week treatment of HCV genotype 1 infection in patients showing RVR [12][31][32], 1 study that compared rapid responders with slow responders [33]. Furthermore, we excluded 1 letter [34], 5 studies on long-term therapy with standard interferon [35][36][37][38][39], 3 studies on HCV/HIV co-infected patients [40][41][42], 2 non-randomized studies [43][44], 1 study in which patients in the extended therapy group were treated with low dose peginterferon or ribavirin [45]. We also excluded 1 study on slow responders with HCV genotype 2/3 infection, 1 study that treated the slow responders by administration of triple therapy with histamine as a part of or throughout the subjects’ treatment duration [46], and 1 study with patients randomized at the baseline but not according to RVR and EVR [47]. Ultimately, 7 studies that met our criteria were included [48][49][50][51][52][53][54].

### 4.2. Included Studies

One study was from Japan, 2 from Spain, and one from each of Austria, the US, Italy, and Germany. All studies were published as full texts in peer-reviewed journals from 2005 to 2010. One study, by Sanchez-Tapias et al., was partially randomized. Methodological quality factorsincluding random sequence generation and allocation concealment were not provided in the reports by Ferenci et al., Miyase et al., and Berg et al. One report by Miyase et al. was in Japanese. We excluded some patients from the relevant studies. From the study reported by Ferenci et al., 262 subjects were excluded because they showed RVR or because of the lack of randomization in the study for the duration of therapy, and 28 subjects were excluded because of infection with HCV genotype 4. 35 subjects with non-genotype 1 infection and 184 with RVR were excluded from the Sanchez-Tapias study and 185 subjects with RVR and 128 patients who were not slow responders were excluded from the study reported by Mangia et al. From the study reported by Berg et al. 81 subjects were excluded because they showed RVR and 168 were excluded because of lack of information about whether they were slow responders. Ultimately, 1206 subjects, 631 of whom received extended therapy, were included in the meta-analysis. SVR was similarly defined across all studies as the lack of RVR along with achieving an undetectable PCR or at least a 2-log decrement in the HCV-RNA levels from baseline after 12 weeks or undetectable HCV-RNA levels after 24 weeks. Table 1 shows some characteristics of the included studies. All trials had similar inclusion criteria. Chronic hepatitis C was diagnosed on the basis of the presence of HCV-RNA in the blood, elevated plasma transaminase levels for at least 6 months, and evidence of chronic viral hepatitis on performing a pretreatment liver biopsy. The exclusion criteria were also very similar in all the trials and consisted of decompensated liver disease; autoimmune mediated diseases; chronic hepatitis B; significant co-morbidities such as HIV, kidney disease, cardiovascular disease, psychiatric illnesses, or history of hospitalization for major depression; poorly controlled diabetes; prior organ transplantation; seizures or brain injury requiring medication for stabilization; or hematological diseases with anemia, low platelet count, or neutropenia. Pregnant or breast-feeding women were also excluded from the study.

**Table 1:** Characteristics and Results of Methodological Quality Assessment of the Included Studies

Authors	Origin ofSamples	Randomization	Allocation Concealment	Naive	Type of Peginterferon
Mangia et al., 2008, [48]	Italy	Adequate	Adequate	Yes	2a/2b
Berg et al., 2006, [49]	Germany	Unclear	Unclear	Yes	2a
Ferenci et al., 2010, [50]	Austria	Unclear	Unclear	Yes	2a
Pearlman et al., 2007, [51]	US	Adequate	Adequate	Yes	2b
Sanchez-Tapias et al., 2006, [52]	Spain	partially	Inadequate	Yes	2a
Miyase et al., 2010, [53]	Japan	Unclear	unclear	NR a	2b
Buti et al., 2010, [54]	Spain	Adequate	Adequate	Yes	2b

^a^ Abbreviation: NR, not reported

### 4.3. Characteristics of the Patients

Patients’ characteristics are presented in Table 2. Miyase et al. reported their patients’ baseline data according to the virological responses but not according to the treatment groups. Sanchez-Tapias et al. did not report the percentages of patients with extensive fibrosis (stage F3/F4), and Pearlman did not report the patients’ baseline levels of liver enzymes. In the study by Mangia et al. 44% of the total studied patients were excluded, so the data provided by the authors could not be considered representative of the characteristics of the included patients.

**Table 2:** Characteristics of the Patients in the Included Studies^a^

Authors	Men, %		Age, y		ALT b, IU/L		Viral load, IU/mL		Cirrhosis, F3/F4, %	
	72 wk	48 wk	72 wk	48 wk	72 wk	48 wk	72 wk	48 wk	72 wk	48 wk
Ferenci et al.	65	64	44.3 ± 10.2 c	45.1 ± 10.6 c	93.3 ± 62.7 c	91.9 ± 74.7 c	700 (K d)	650 (K)	19	20
Pearlman et al.	65	67	54	56	NR b	NR	5400 (K)	5300 (K)	25	27
Nagaki et al.	67	62	54	62	52	64	≥ 1500 (K) 67%	≥ 1500 (K) 77%	77	62
Sanchez-Tapias et al.	63	69	43.2 ± 10.2 c	42.8 ± 9.9 c	2.7 ± 1.6 c ULN b	2.4 ±1.3 c ULN	1110 ± 1333 c (K)	963 ± 1153 c (K)	NR	NR
Buti et al.	63	60	46.5 ± 11.6 c	44.5 ± 9.9 c	85 ± 71 c	76 ± 48 c	> 800 (K) 93.2%	> 800 (K) 87.2%	NR	NR
Miyase et al.	33		58		83		2243 (K)		NR	

^a^ In the study by Mangia et al. 44% of the patients were excluded, so the data provided by authors could not be considered representative for the characteristics of the included patients.

^b^ Abbreviations: ALT, Antiretroviral therapy; NR, Not reported; ULN, Upper limit of normal

^c^ Value ± SD

^d^ K: 103

### 4.4. Comparison of SVR Achieved during the 72-week Therapy with that Achieved with the Standard 48-week Therapy

**Figure 2:**
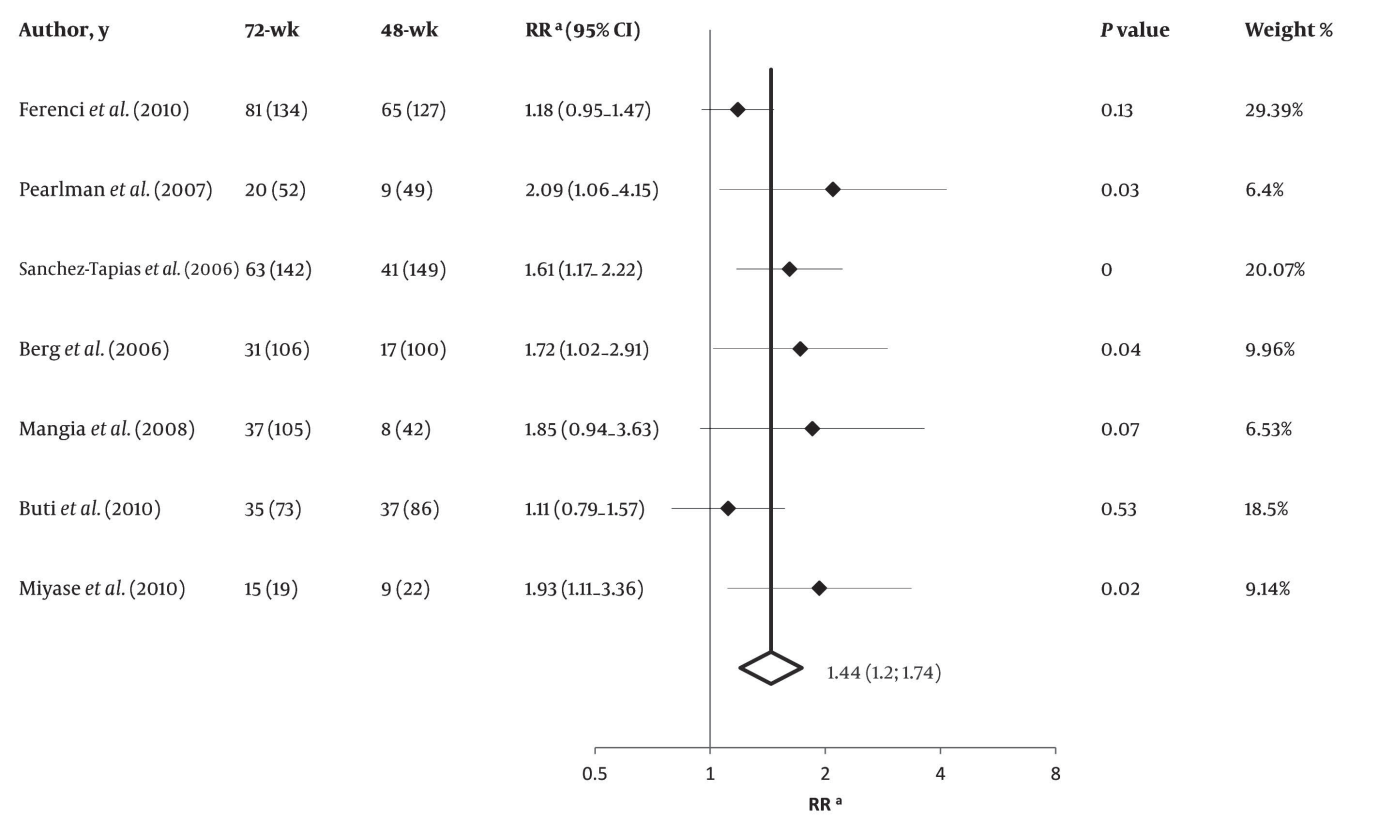
Summary Estimate with 95% Confidence Interval for Relative Risk (RR) of Sustained Virological Responses (SVR) Rates Achieved with 72- vs. 48-week Treatment of Hepatitis C Virus (HCV) Infection with Peginterferon and Weight-Based Ribavirin

**Figure 3:**
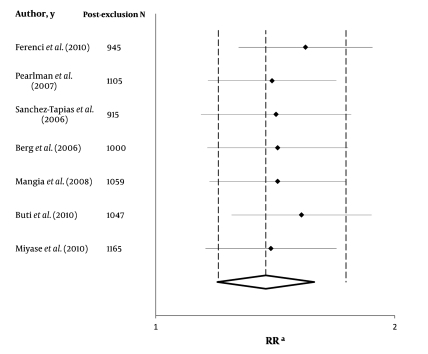
Exclusion Sensitivity Plot

**Figure 4:**
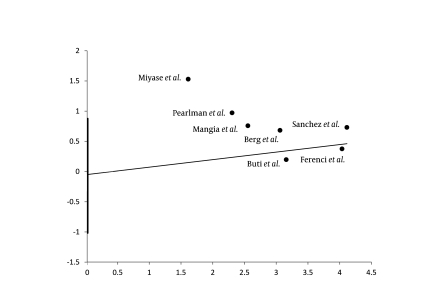
Harbord’s Regression Line for Publication Bias Assessment

The slow virological responders who received the 72-week anti-HCV therapy had a significantly higher likelihood of achieving SVR than their counterparts who received the standard 48-week anti-HCV therapy [RR = 1.44 (95% CI 1.20–1.74)]. The heterogeneity in funnel plots was not significant (P = 0.19), and the actual heterogeneity between study results that could not be justified by chance alone was 30% (I2). Furthermore, Tau-squared value was 0.01 (95% CI, 0–0.09). Figure 2 shows summary estimates with their 95% CI for each study along with the weighted-pooled estimate. Figure 3 shows results of the exclusion sensitivity analysis. The pooled result was evidently robust and did not rely on any single study result. The assessment of publication biases by using Harbord’s modified test showed a non-significant P value (P = 0.11). Moreover, the P value for Egger’s test was 0.59. Figure 4 shows Harbord’s regression line.

### 4.5. Comparison of the Safety of the 72-week Therapy with that of the Standard 48-week Therapy

The information about the safety profiles of the 72week therapy vs. the standard 48-week therapy was not well elucidated. Table 3 shows the pooled estimate of comparative safety and number of patients included in the analyses. Due to the low number of patients and lack of data on adequate reporting of safety, the evidence on the comparative safety of the 72-week and 48-week therapies is not robust. Although none of the differences in the individual adverse effects reached statistical significance, the number of patients who discontinued the therapy due to safety concerns or voluntarily was considerably greater among the patients who received the 72-week therapy.

## 5. Discussion

In this systematic review, we assessed the comparative efficacy of anti-HCV therapy with peginterferon alpha and weight-based ribavirin for durations of 72 weeks and 48 weeks in slow virological responders with HCV genotype 1 infection who were seronegative for HIV and HBV co-infections. Our results suggest that the 72-week treatment helped in achieving a significantly higher SVR rate than the 48-week treatment in slow virological responders with HCV genotype 1 infection. Because of the high mean viral load of the included patients, our findings can easily be extrapolated to the slow virological responders with high viral load of HCV genotype 1 or hard to treat HCV infections. Although the differences among any individual adverse effects were not statistically significant, voluntary treatment discontinuation or discontinuation because of safety reasons was significantly greater in the 72-week treatment group. This finding can be attributed to longer or severer adverse effects in the extended therapy group. The SVR rate achieved with the 72-week therapy can be increased by encouraging the patients to complete the 72-week therapy, closely following-up the patients’ condition, and reducing the treatment discontinuations by managing the adverse effects.

We are highly confident of the effectiveness of our finding for the following reasons: [1] we considered nonsignificant heterogeneity test results despite clinical heterogeneity across studies including (a) different treatment protocols for ribavirin therapy, (b) inclusion of some patients with previous history of treatment, (c) different peginterferon type, and (d) different ethnicity of the subjects; [2] we found robust results that were confirmed by exclusion sensitivity analysis, which showed that the final estimate was not dependent on any single study results (Figure 4); and [3] we performed non-significant publication assessment. The empirical evidence suggests that trials that fail to refute the null hypothesis have low odds of being published, especially those that are not funded by the industry [55][56]. Our publication bias assessment did not revealed such finding. Trim-and-fill method used to account for the missing data showed results similar to the findings of our original pooled estimate [RR = 1.29 (95% CI 1.13–1.47)] [4]. Of seven included studies, 6 studies had complete and 1 had partial randomized design. Moreover, the robust results of our sensitivity analysis suggest that both naive and treated patients can benefit from the extended therapy.

The greatest limitation in this review was insufficient reporting of safety data and methodological qualities of the RCTs. Therefore, we recommend that investigators of future trials adhere to the Consolidated Standards for Reporting of Trials (CONSORT) to improve the quality of trial reports [57]. Another limitation of our work was that the long-term clinical advantage of extended anti- HCV treatment for HCC, decompensated liver disease, and cirrhosis could not be clarified. Furthermore, there are some concerns about the validity of our findings outside of a clinical trial setting. Our analysis showed that the 72-week treatment course is difficult to tolerate for most patients and there is a high likelihood of premature withdrawal. The odds ratio of 2.7 for voluntary therapy discontinuation in Table 3 supports this perspective. Therefore, in routine clinical practice and outside of a clinical trial setting, this rate of treatment discontinuation can become even higher to an extent that can reduce the rate of SVR. Low external validity of the findings of this review cannot be attributed to the reliability of our findings, but to the nature of the clinical trials included in the review. We suggest that making decision to extend the treatment course should be made on an individually basis and according to the patients’ tolerance during the first 48 weeks of therapy. Although the number of patients who discontinued the treatment because of safety reasons or personal causes was high in the extended therapy group, our meta-analysis showed that the 72-week therapy with peginterferon and ribavirin is significantly superior to the standard 48-week therapy for slow responders with genotype 1 infection.

**Table 3:** Comparative Safety Profile of the 72-Week and 48-Week Anti-HCV Therapies with Peginterferon and Ribavirin

Adverse event	Patients, No.	OR (95% CI)	Heterogeneity Assessment		
			Q (K)	P	I 2
Neutropenia/leukopenia	1571	1 (0.71–1.41)	1.34 (5)	0.85	0%
Thrombocytopenia	326	0.61 (0.29–1.25)			
Fever	485	1.03 (0.69–1.54)	0 (1)	0.96	0%
Asthenia	485	0.96 (0.66–1.38)	0.06 (1)	0.81	0%
Headache	485	1.06 (0.72–1.56)	0.21 (1)	0.65	0%
Flu-like symptoms	485	0.89 (0.51–1.56)	1.85 (1)	0.17	46%
Anemia	1412	1.06 (0.72–1.56)	2.7 (3)	0.44	0%
Depression	615	0.95 (0.12–7.39)	2.14 (1)	0.14	53%
Voluntary therapy discontinuation	1571	2.70 (1.60–4.56)	4.15 (4)	0.39	4%
Therapy discontinuation due to safety reasons	1470	1.54 (1.11–2.14)	0.22 (3)	0.97	0%
